# Impact of *APOE4* Allele on Cell‐Cell Communication and α‐Synuclein Aggregation in Lewy Body Dementia: Insights from Spatial Transcriptomics

**DOI:** 10.1002/alz.092756

**Published:** 2025-01-03

**Authors:** Yunjung Jin, Alexander Q. Wixom, Zonghua Li, Yuanhang Liu, Shunsuke Koga, Hiroaki Sekiya, Hannah Santhakumar, Guojun Bu, Dennis W. Dickson, Na Zhao

**Affiliations:** ^1^ Mayo Clinic, Jacksonville, FL USA; ^2^ Mayo Clinic, Rochester, MN USA

## Abstract

**Background:**

The aggregation of α‐synuclein protein, encoded by the *SNCA* gene, forms Lewy bodies (LBs) in neurons and is a key pathological feature of Lewy body dementia (LBD). Interestingly, the apolipoprotein E gene (*APOE*), primarily expressed in glial cells, is the strongest genetic modifier for LBD. The ε4 allele of this gene (*APOE4*) notably increases the risk of LBD. However, the specific mechanisms through which APOE‐regulated glial cells affect α‐synuclein aggregation in neurons are not yet fully understood.

**Method:**

To explore this, we conducted spatial transcriptomics using the 10X Genomics Visium CytAssist platform on Formalin‐Fixed Paraffin‐Embedded tissue slides from the temporal cortex of human LBD brains (N = 4 brains in controls and N = 6 brains in LBD group, with an equal ratio of *APOE3/3* and *APOE3/4* genotypes). We defined the neuronal layers of the gray matter (Layers 1 to 6, L1‐6) and white matter in each brain based on the gene signature of each spot. We also identified LBs from hematoxylin and eosin (H&E) staining on the same slides, and annotated LB‐positive (LB+), LB‐surrounding and LB‐negative (LB‐) spots in each brain using Loupe Browser. We evaluated *SNCA* and *APOE* gene expression in each layer and analyzed cell‐cell communication using the CellChat tool across the layers.

**Result:**

We observed that the *SNCA* gene was highly expressed in L5, which corresponded to the areas where most LBs were found. Notably, within LB+ spots across all layers, both *SNCA* and *APOE* gene expression levels were elevated in *APOE3/4* cases compared to *APOE3/3* cases. Furthermore, we identified cell‐cell communication networks between LB+ and LB‐surrounding spots, particularly in L4 and L5. These networks were enriched by microglia‐related signaling pathways, including APOE signaling, migration inhibitory factor (MIF) signaling, and colony stimulating factor 1 (CSF1) signaling. Interestingly, *APOE3/4* brains exhibited significantly reduced interactions and lower interaction strength in these networks compared to *APOE3/3* samples.

**Conclusion:**

Our findings indicate that impaired cell‐cell communication, such as microglia‐mediated immune responses around LB regions, may be associated with the increased risk presented by the *APOE4* allele in LBD brains.

